# The role of pyroptosis-related lncRNA risk signature in ovarian cancer prognosis and immune system

**DOI:** 10.1007/s12672-023-00767-3

**Published:** 2023-08-19

**Authors:** Yanling Wu, Lei Liang, Qin Li, Lilu Shu, Peter Wang, Shufeng Huang

**Affiliations:** 1 Department of Gynecology, Shenzhen Qianhai Shekou Free Trade Zone Hospital, Shenzhen, 518067 Guangdong China; 2 Department of Research and Development, Zhejiang Zhongwei Medical Research Center, Hangzhou, 310018 Zhejiang China

**Keywords:** LncRNA, Ovarian cancer, Target, Pyroptosis, Immune system

## Abstract

Ovarian cancer is a leading cause of death in females with gynecologic cancers. Pyroptosis is a relatively new discovered programmed cell death that is believed to be associated with inflammation. However, studies on pyroptosis-related lncRNAs in ovarian cancer are limited. In this study, we identified 29 pyroptosis-related genes and screened out 72 pyroptosis-related lncRNAs. Furthermore, the 72 lncRNAs were eliminated to 2 survival-related lncRNAs using Cox regression and Lasso regression to build an ovarian cancer prognostic prediction signature and were further validated on the test set. We adopted a riskscore from the two-gene signature, and the survival in low-risk group was higher than the high-risk group. Functional enrichment analysis indicated that the differentially expressed genes (DEGs) between two risk groups were associated with tumor immunity. This study implies that pyroptosis-related genes are closely related to tumor immunity and could be potential therapeutic factors for ovarian cancer treatment.

## Introduction

Ovarian cancer is one of the most common gynecologic cancers in the United States in 2023, which is also a leading cause of death in female reproductive system [1]. There is no efficient pre-screening methods for detecting early stages of ovarian cancer so far, therefore most of the ovarian cancer patients were diagnosed at late stages [2]. The common treatments for ovarian cancer are surgery and chemotherapy, but due to relapse or drug resistance, especially for patients in stages III and IV, the prognosis of patients with ovarian cancer is not optimistic [3]. The 5 year survival rate of ovarian cancer patients in stage I is higher than 90%, compare to the 5 year survival rate of 70%, 39% and 17% of ovarian cancer patients in stage II, III and IV, respectively [4].

The alternative treatment for cancer patients is the immunotherapy, which is to identify certain biomarkers that could trigger the patient immune system to eliminate cancer cells [5, 6]. Immunotherapy could stimulate peripheral immune response, enhance local and systemic immunity. For instance, targeting PD-1 (nivolumab, pembrolizumab), CTLA-4 (tremelimumab, ipilimumab) and PD-L1 (avelumab, durvalumab, aterolizumab) has been reported to treat ovarian cancer patients [7–9]. Programmed cell death (PCD) includes apoptosis, autophagy, necroptosis, pyroptosis, ferroptosis and cuproptosis [10, 11]. Pyroptosis is a form of PCD that is triggered by caspase-1/4/5 in humans, which is also closely related to inflammation [12, 13]. Caspase-1 is a key enzyme to mediate the process of pyroptosis. Pyroptosis exhibits plasma-membrane impair and leads to release of proinflammatory intracellular factors such as IL-1b [14, 15]. The studies indicate that the members of gasdermin (GSDM) family are executors of pyroptosis [16]. For example, caspase-1 stimulates the cleavage of gasdermin D (GSDMD) and thus triggers pyroptosis [17]. Several studies have confirmed the relationship between pyroptosis and inflammatory response in gynecological tumors [18, 19]. One study described the different roles of pyroptosis, necroptosis and ferroptosis in the development of ovarian cancer [20]. Another study reported differential expression and copy number variation of GSDM family members in pyroptosis in serous ovarian cancer [21]. Moreover, it has been documented that pyroptosis-related genes are linked to tumor infiltration in ovarian cancer [22]. Prior research indicated that the pyroptosis-related genes involve in the tumor immunity and have a prognostic value in ovarian cancer, that absent-in-melanoma-2 (AIM2) acts as a cancer-promoting gene in ovarian cancer, but in other cancers, AIM2 is a tumor suppressor; and the high expression of gasdermin A (GSDMA) and phospholipase C gamma 1 (PLCG1) in ovarian cancer was connected to poor survival in patients [23, 24]. The inflammation is the two sides of a coin, in that it could both promote or inhibit the tumor development in various cancers, so does the pyroptosis [25].

Numerous studies have demonstrated that long non-coding RNA (lncRNA) plays a major role in regulatory functions in carcinogenesis [26–29]. LncRNAs are the RNAs that have length longer than 200 nt, and previously was believed to be transcriptional noise [30]. For example, GAS5 inhibits ovarian cancer development by inducing inflammation response [31, 32]. Cumulative studies have investigated the function of lncRNAs in tumorigenesis via regulation of multiple biological processes, including but not limited to proliferation, migration, drug resistance, cell signaling, and metastasis in cancer cells [33–36]. Both pyroptosis-related genes and lncRNAs are playing a substantial role in ovarian cancer development, but the research on the prognostic value of pyroptosis-related lncRNAs is still limited [37]. The aim of this study is to build a prognostic prediction model with pyroptosis-related lncRNAs by stratifying ovarian cancer patients into different risk groups, so that we could build a promising risk signature model. The tumor microenvironment has been identified to be associated with ovarian cancer occurrence, metastasis and progression [38, 39]. Therefore, on top of the prediction model, we’ve also investigated the relationship between the risk signatures and tumor immune infiltration and microenvironment in ovarian cancer.

## Methods

### Data collection and preprocessing

Ovarian cancer RNA sequencing data of the tumor tissues (n = 381) and clinical data (n = 608) were obtained from the Cancer Genome Atlas (TCGA) database. We also downloaded the gene expression data of the ovarian normal tissue from the pooled Genotype-Tissue Expression (GTEx, n = 82) database. We excluded genes with zero expressions across all of the patients, and patients with no survival data, which led to 378 samples in the subsequent survival analysis. The pyroptosis-related genes were collected from literature [40], and of the 33 pyroptosis genes, only 29 were identified in our merged data with TCGA and GTEx. We identified pyroptosis-related lncRNAs through the Pearson correlation between pyroptosis-related genes and lncRNAs (|r| > 0.5, p < 0.001).

### Networks and differential expression analysis

A protein-protein interaction (PPI) network of pyroptosis genes was constructed with the software STRING (score > 0.9). Meanwhile, a co-expression network of pyroptosis genes and pyroptosis-related lncRNAs were created in the software Cytoscape. We identified the differentially expressed genes (DEG) with the “limma” and “edgeR” R packages between the tumor tissues and the normal tissues in pyroptosis genes (|logFC| >=1 and p < 0.05).

### Construction and validation of a prognostic signature

To build a prognostic model, we first randomly split a total of 378 TCGA samples into a training set (n = 264) and a test set (n = 114), at a ratio of 7:3, so as the corresponding clinical data. Prognostic pyroptosis-related lncRNAs were obtained from univariate Cox proportional regression analysis (p < 0.05). And we further selected the lncRNAs with Lasso regression and multivariate Cox proportional regression (p < 0.05). We incorporated the 10-fold cross validation in Lasso regression to find the appropriate λ for the further feature selection. With the selected prognostic lncRNAs, we calculated the riskscore using the following equation, where n is the number of lncRNAs, Expi is the expression of lncRNAs and Coefi is the coefficient in the multivariate Cox regression. We then categorized them into high and low risk groups based on the median value of the riskscore. Thus, a prediction model of prognostic signature was built with the selected lncRNAs and survival data. The Kaplan Meier curve and log-rank test were used to compare the survival times between high- and low-risk groups. The training set was used to establish the foundation of the prediction model, and the test set was used to evaluate the overall performance of the model. The model was assessed using AUC in both training and test sets. Nomogram was also used to evaluate the clinical benefits in risk signature.

$$\mathop \sum \limits_{{i = 1}}^{n} Expi \cdot Coef{\text{i}}$$

### Functional enrichment analysis and immune infiltration analysis

The 264 ovarian cancer patients in the training set were stratified as high and low risk groups based on the prognostic signature. To investigate the biological functions and pathways carried by the genes, we performed functional enrichment analysis on risk group DEGs. The “limma” and “clusterProfiler” R packages were used to identify the DEGs and to perform Gene oncology (GO) enrichment and Kyoto Encyclopedia of Genes and Genomes (KEGG) analyses, with |log2FC| >= 2 and p < 0.05. The “gsva” R package was then used to calculate the score for 22 immune cells and 12 immune functions. The ssGSEA scores were compared between high and low risk groups in both training and test sets. Further, we explored the differences in immune cell infiltration using the CIBERSORT algorithm.

### Statistical analysis

**Fig. 1:**
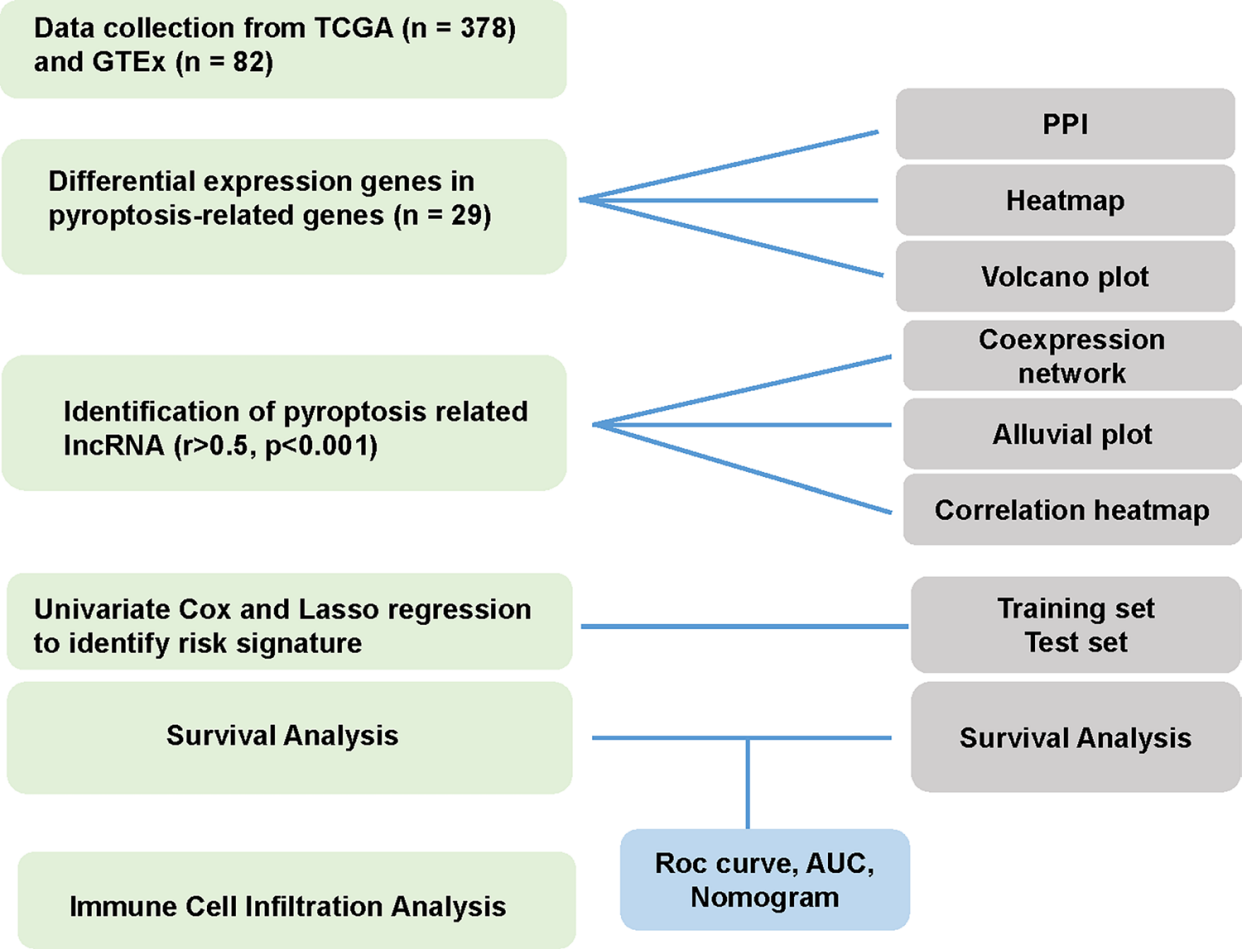
Flowchart of the data analysis in this study

All the analyses were conducted in R software (4.2.1). Statistical methods and data processing were descripted in detail in the above section. In general, *P* value less than 0.05 is considered as significant in this study. The overall workflow of this study is summarized in Fig. 1.

## Results

### Pyroptosis-related lncRNAs in ovarian cancer patients

**Fig. 2:**
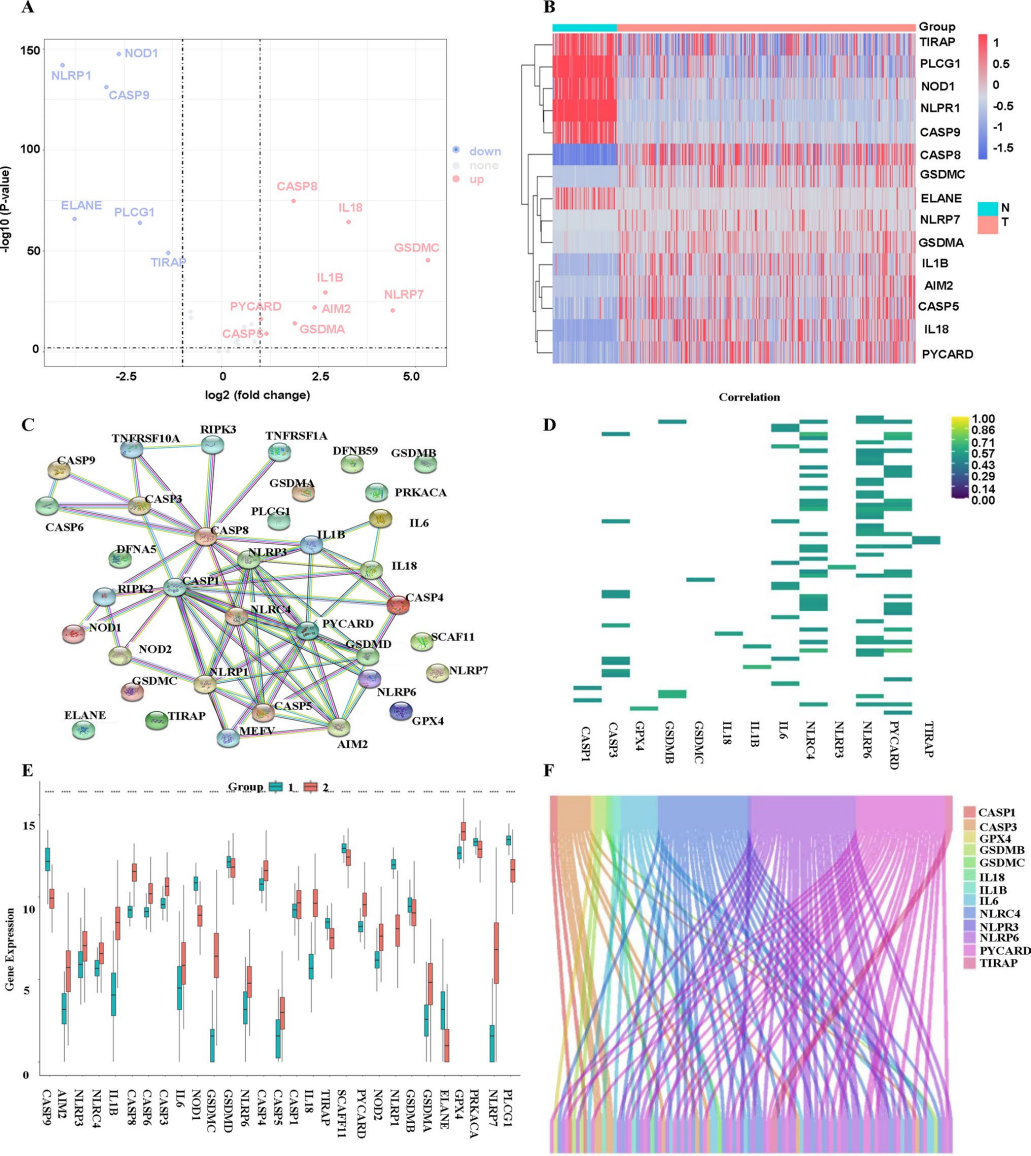
Networks and differential expression analysis of pyroptosis-related genes. **A** Volcano plot of the differential expression analysis of the pyroptosis related genes, where red represents upregulated genes and blue represents downregulated genes. **B** Heatmap of upregulated and downregulated pyroptosis-related genes in normal and tumor ovarian tissues. **C** Network of 29 pyroptosis-related genes. **D** The heatmap of the correlation of pyroptosis-related genes and lncRNAs, all with Pearson correlation |r| > 0.5, p < 0.05. **E** The boxplot of gene expression difference between normal and tumor tissues in pyroptosis-related genes. **F** An alluvial plot of pyroptosis-related genes and lncRNAs.

From the pyroptosis-related genes, we recognized 9 upregulated genes, including CASP8 (caspase 9), IL18 (interleukin 18), GSDMC (gasdermin C), NLRP9 (NLR family pyrin domain containing 9), GSDMA, AIM2, IL1B (interleukin 1 beta), PYCARD (PYD and CARD domain containing) and CASP5 (caspase 5), and 6 downregulated genes, such as NLRP1 (NLR family pyrin domain containing 3), NOD1 (nucleotide binding oligomerization domain containing 1), CASP9, ELANE (neutrophil elastase), PLCG1 and TIRAP (TIR domain containing adaptor protein), as shown in Fig. 2A, all with |logFC| >=1 and *P* < 0.05, produced by the “limma” and “edgeR” packages. The expression levels showed differences in gene expression of the pyroptosis genes between the normal and tumor ovarian tissues (Fig. 2B). The protein-protein interaction of the 29 pyroptosis genes was shown (Fig. 2C), where the network only included protein interactions with an interaction score greater than 0.9. The 72 pyroptosis-related lncRNAs were selected using Pearson correlation, and we calculated the correlation and *P* value between 16,754 lncRNAs and the 29 pyroptosis-related genes (|r|>0.5 and *P* < 0.001, Fig. 2D-F). It also showed that gene expression of pyroptosis-related genes was all significantly different between normal and tumor groups (Fig. 2E)

### Construction of the prognostic signature

**Fig. 3:**
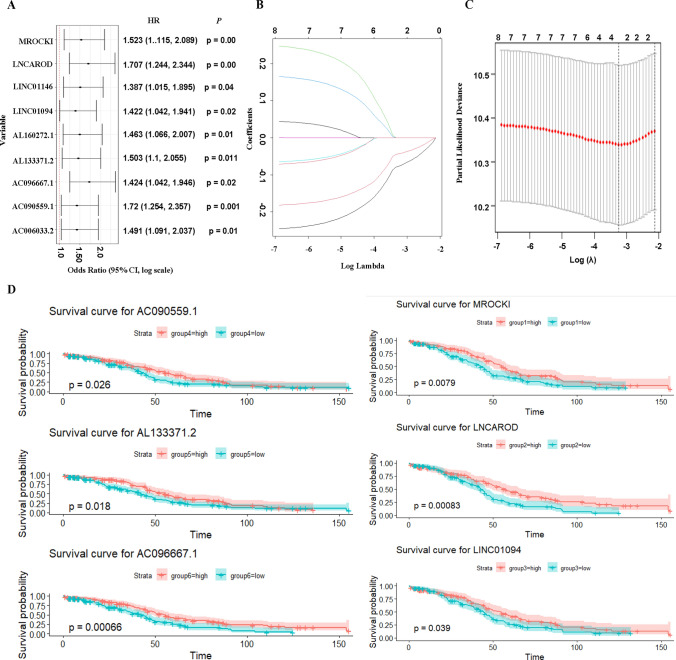
Survival-related lncRNAs selection. **A** A forest plot of Univariate Cox regression coefficients that are correlated with survival time. **B** Plot of lambda selection in Lasso regression algorithm. **C** Lasso-Cox regression of pyroptosis survival-related lncRNAs. **D** Kalplan-Meier curves for lasso selected lncRNAs

The summary statistics of the ovarian cancer patient were presented in Table 1. From the table, we found that the average age of ovarian cancer patient who were dead is older than the patients who are alive. With the 72 selected pyroptosis-related lncRNAs, we performed univariate Cox regression on the corresponding clinical data. Furthermore, the majority of the patients are Caucasians, and the ovarian cancer was mainly concentrated at stage III. Based on the *P* values of each univariate Cox regression, 9 survival-associated pyroptosis-related lncRNAs with *P* < 0.05 were selected for further analysis, which were shown on the forest plot (Fig. 3A). Lasso regression was used for further feature selection. With 10-fold cross validation in Lasso regression, the best performed lambda was selected for the final lasso regression parameter (Fig. 3B, C). Two survival-related lncRNAs were selected from the Lasso regression model. We then applied multivariate Cox regression to build potential risk signature for our final survival-related pyroptosis lncRNAs, which is the foundation of our risk model. The Kaplan-Meier curves of the lncRNAs were shown (Fig. 3D).

**Table 1:** The summary statistics of the ovarian cancer patient were presented

Items	Alive (N = 146)	Dead (N = 232)	Overall (N = 378)
Age (years)			
Mean (SD)	57.2 (11.6)	61.1 (11.0)	59.5 (11.4)
Median (Min, Max)	57.0 (30.0, 87.0)	60.0 (36.0, 87.0)	59.0 (30.0, 87.0)
Race			
Indian/native	0 (0%)	2 (0.9%)	2 (0.5%)
Asian	7 (4.8%)	4 (1.7%)	11(2.9%)
Black	8 (5.5%)	17 (7.3%)	25 (6.6%)
Hawaiian	0 (0%)	1 (0.4%)	1 (0.3%)
Not reported	8 (5.5%)	3 (1.3%)	11 (2.9%)
White	123 (84.2%)	205 (88.4%)	328 (86.8%)
Stages			
Stage I	0 (0%)	1 (0.4%)	1 (0.3%)
Stage II	17 (11.6%)	5 (2.2%)	22 (5.8%)
Stage III	111 (76.0%)	183 (78.9%)	294 (77.8%)
Stage IV	16 (11.0%)	42 (18.1%)	58 (15.3%)
Missing	2 (1.4%)	1 (0.4%)	3 (0.8%)

### Distribution of gene expression in different groups

**Fig. 4:**
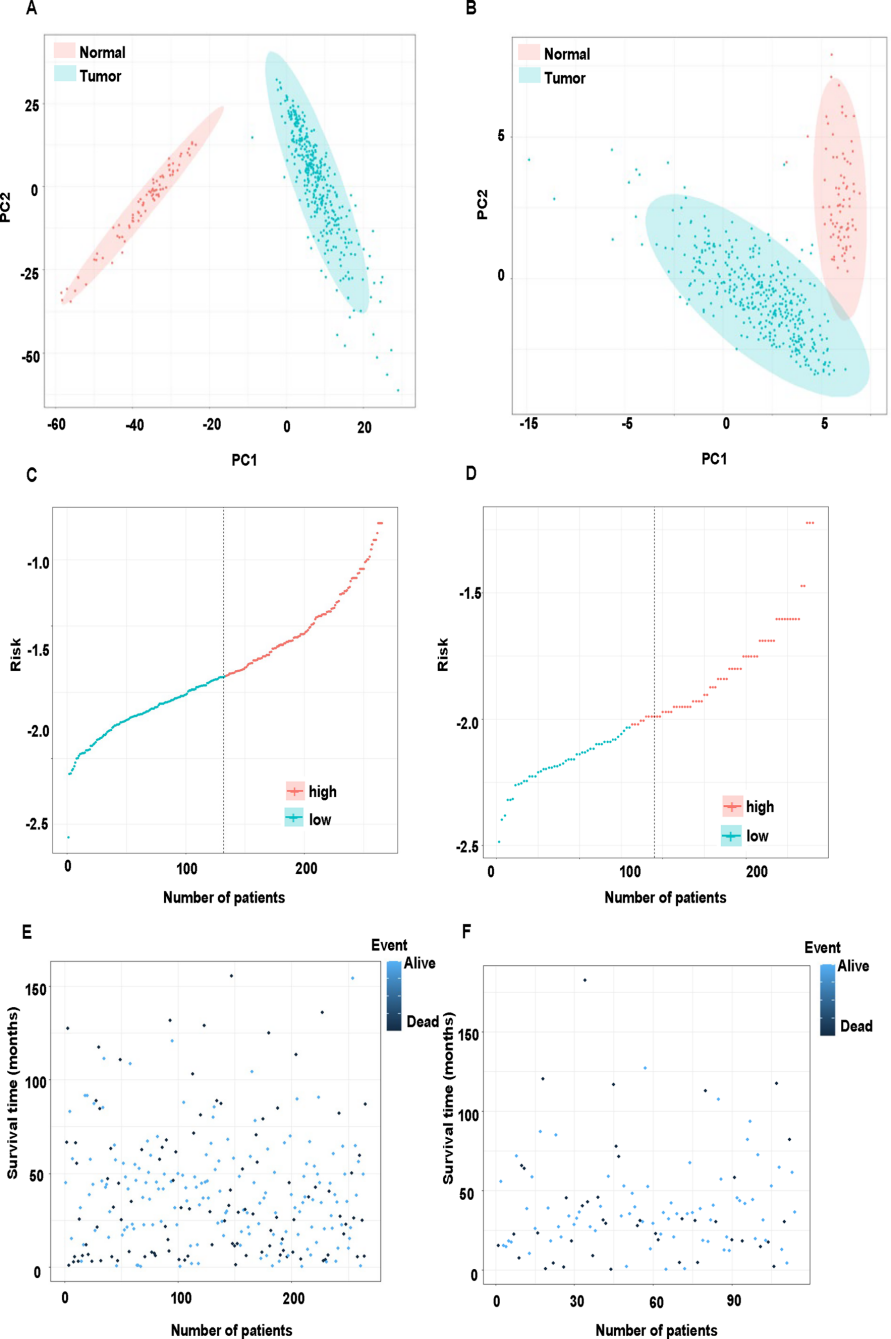
Distribution of gene expression in different groups. **A** PCA of all lncRNAs in tumor and normal tissues. **B** PCA of pyroptosis genes in tumor and normal tissues. **C** Distribution of patients who are classified as high and risk groups based on riskscore in the training set. **D** Distribution of patients who are classified as high- and low-risk groups based on riskscore in the test set. **E** Distribution of patients who are alive or dead in the training set. **F** Distribution of patients who are alive or dead in the test set

The risk score was calculated with the equation described in Methods section. We categorized each patient to low- or high-risk groups based on the risk score, that we defined patients with risk score lower than the median of risk score as low-risk group and patients with risk score higher than the median as high-risk group. The principal components analysis (PCA) data showed that normal and tumor tissues in ovarian are distinctly different from each other (Fig. 4A, B). Hence, we were curious about how the riskscore would tell them apart. The distribution of patients in riskscore and vital status was different in high and low risk scores in training and test sets, respectively (Fig. 4C, D). Furthermore, we have also showed how the patients who were dead or alive were distributed, regarding to the survival days in the training and test sets (Fig. 4E, F).

### Univariate and multivariate Cox regression on riskscore

**Fig. 5:**
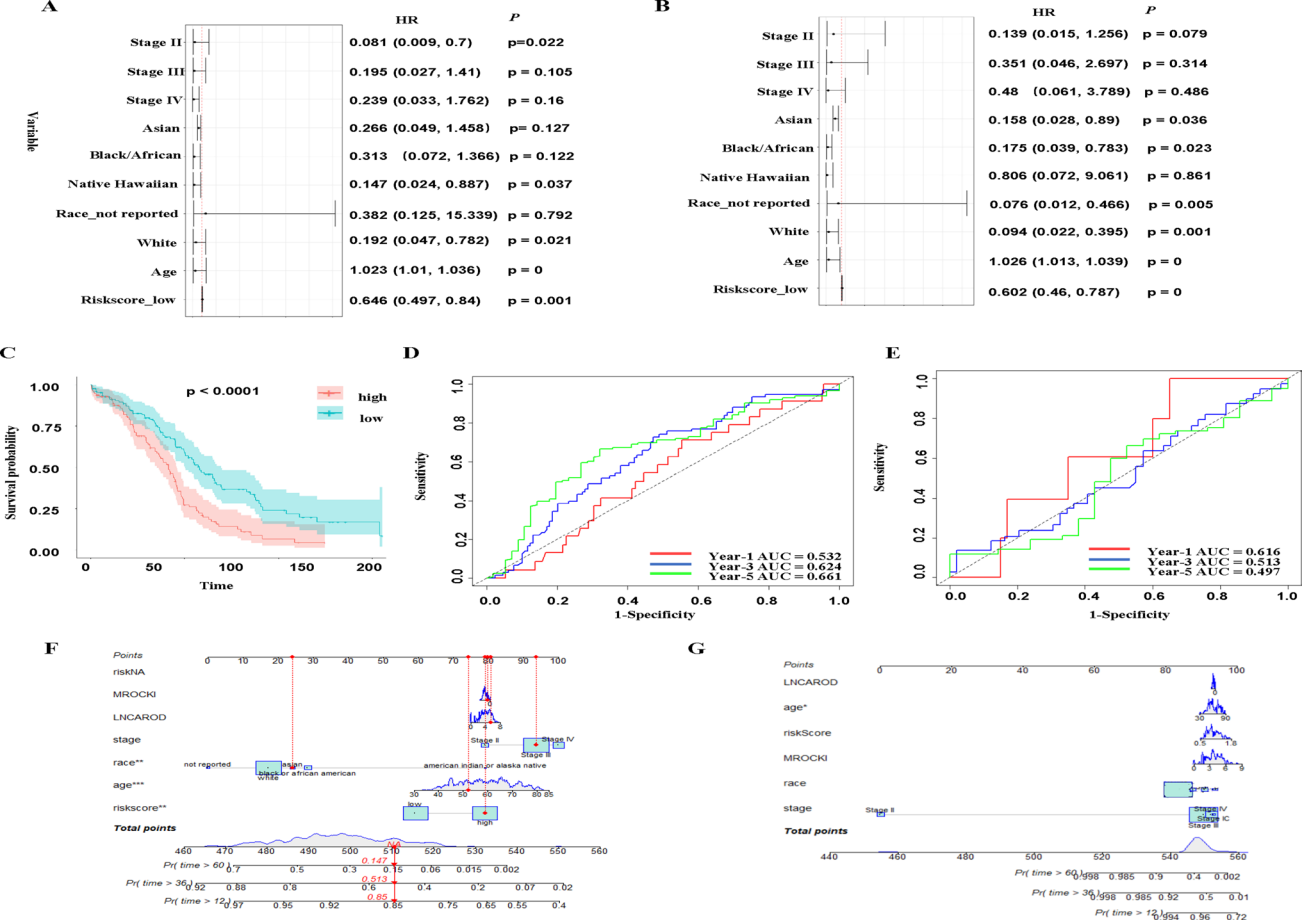
Univariate and Multivariate Cox regression on riskscore. **A** A forest plot of univariate cox regression of riskscore and clinical information. **B** A forest plot of multivariate cox regression of riskscore. **C** Kaplan-Meier curve of riskscore on the training set. **D** ROC curves of riskscore in year-1, year-3, and year-5 on the training set. **E** ROC curves of riskscore in year-1, year-3, and year-5 on the test set. **F** A Nomogram predicting the survival rate at 1 year, 3 year and 5 year on training set. **G** A Nomogram predicting the survival rate at 1 year, 3 year and 5 year on test set

In the meantime, we performed univariate and multivariate cox regression on riskscore and clinical information (age, race, stage), respectively. The *P* value indicated that riskscore is an independent predictor of survival analysis (Fig. 5A, B). The large range of the hazard ratio in the race not reported group might be due to the small sample size in our data. The Kaplan-Meier curve of the grouped riskscore in training set was shown (Fig. 5C). Meanwhile, the receiver operating characteristic (ROC) was used to assess the performance of the risk model. The AUC for year-1 is 0.532, and 0.624, 0.661 for year-3 and year-5, respectively (Fig. 5D). The ROC curves for the test set were shown (Fig. 5E), although the performance of the riskscore in prediction survival is not as good as the training set. At the same time, the clinical information of patients was also assessed through nomogram in both training and test sets (Fig. 5F and G). It showed that based on our riskscore, the survival rate was lower for patients who suffered from ovarian cancer longer, which is as expected.

### Functional enrichment analysis

**Fig. 6:**
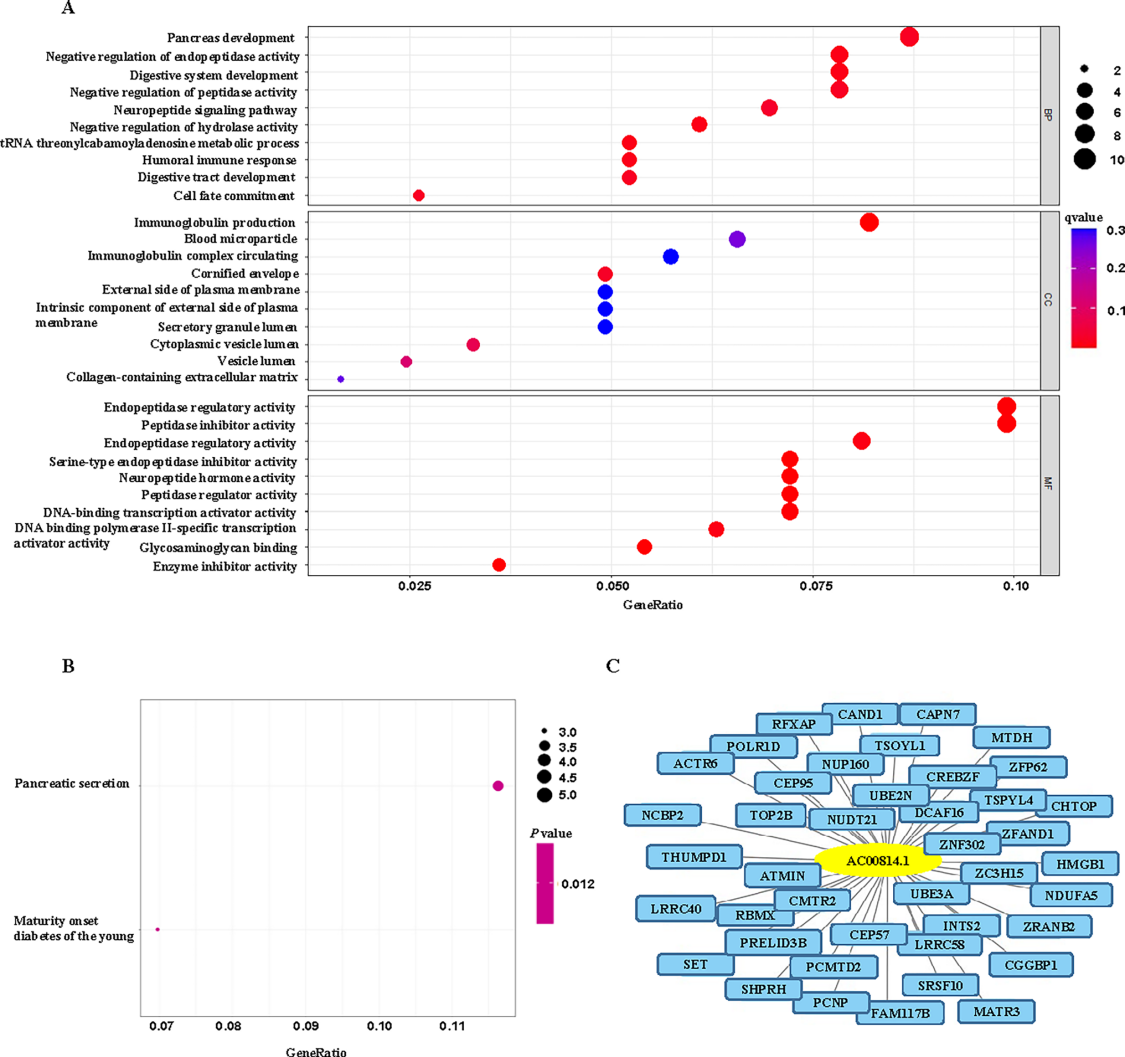
Gene enrichment analysis in risk groups and target genes in pyroptosis-related lncRNAs. **A** GO enrichment analysis in risk groups. **B** KEGG enrichment in risk groups. **C** Target genes related to pyroptosis-related lncRNA, AC00814.1

We stratified patients in the training set into high and low risk groups based on the riskscore. The DEG was then applied to the risk groups to perform functional enrichment analysis. The GO enrichment on the stratified riskscore DEGs demonstrated that genes were enriched in several biological processes, such as digestive development, regulation of peptidase activity and more importantly, immune response (Fig. 6A). DEGs were also enriched in a few immunoglobulin-related cellular components. All of the enrichment suggested that the DEGs based on riskscore were closely related to immune response. KEGG pathways showed that pancreatic secretion and maturity onset diabetes of the young were enriched in the DEGs, where pancreas was also enriched in GO analysis (Fig. 6B). Of all the pyroptosis survival-related lncRNAs, we were only able to identify the target genes of AC00814.1 (Fig. 6C). Future work should be done regarding the potential target genes of this pyroptosis-related lncRNA.

### Immune infiltration analysis

**Fig. 7:**
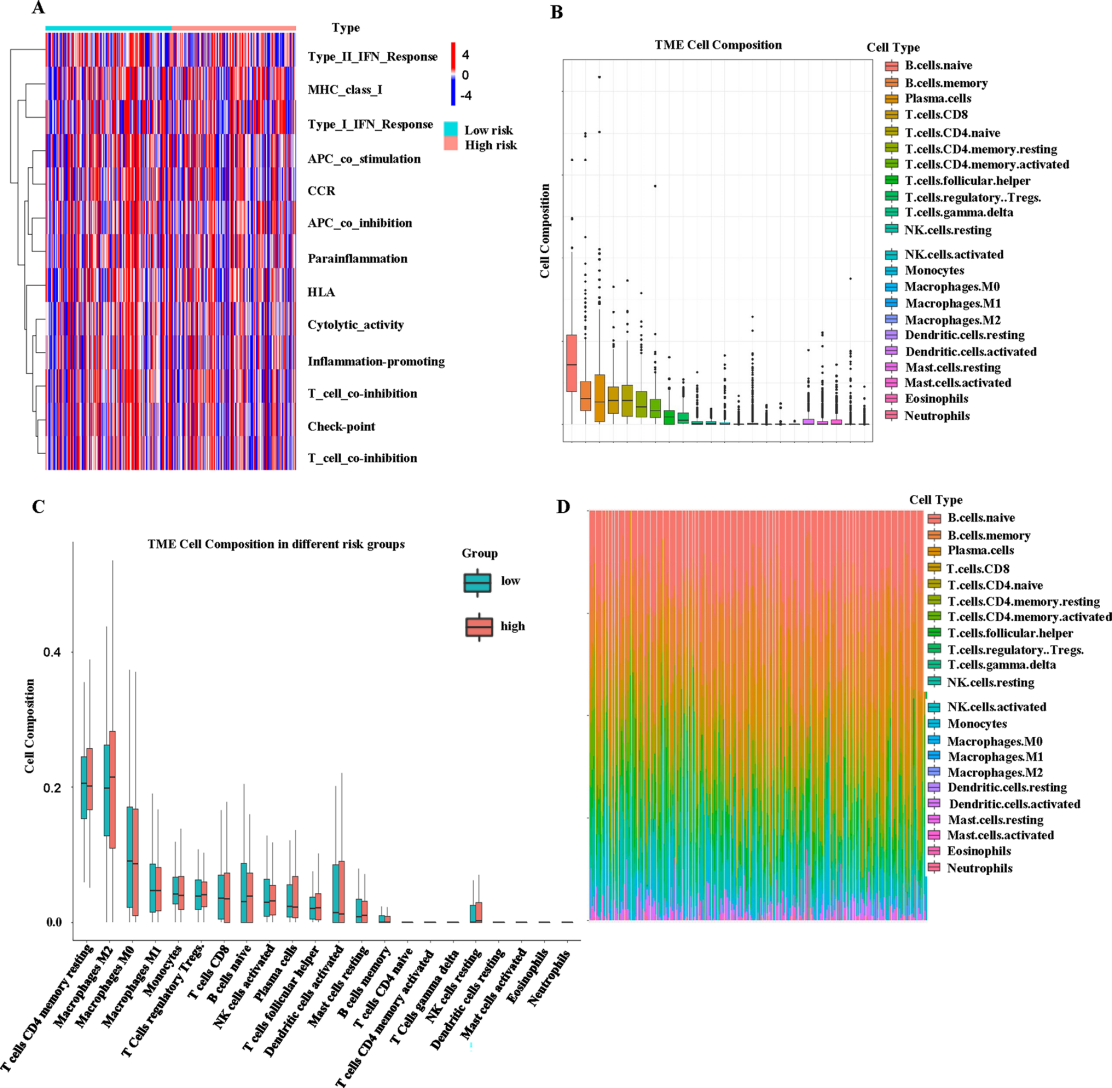
Comprehensive analysis in immune function analysis, immune cell composition and immune infiltration. **A** A heatmap for different immune functions in high- and low-risk groups. **B** A boxplot for different immune cells. **C** A boxplot for different immune cells in high- and low-risk groups. **D** Immune cell infiltration analysis

Further, with the enrichment in immune response from GO analysis, we obtained the estimated enrichment score of the immune cells using “GSVA” R package in the training set, both in low and high risk groups (Fig. 7A). Moreover, we also obtained the estimated enrichment score of the immune cells in the test set. Unfortunately, only three out of 114 patients in the test set were concluded in the algorithm and the three patients were all in the low risk group. Thus, we were unable to perform group comparison in the test set. Furthermore, we explored the differences in immune cell infiltration using the CIBERSORT algorithm in the training set (Fig. 7B). At the same time, we also constructed a boxplot to compare the cell composition differences in immune cell infiltration in high and low risk groups (Fig. 7C). Regrettably, none of the immune cell infiltration in high and low groups were significant in the training set. A barplot showed the percentage of the 22 immune infiltration cells from the training set (Fig. 7D).

## Discussion

In the current study, we detected 72 pyroptosis-related lncRNAs with the specific criterions. We further screened out six survival-related lncRNAs using LASSO and Cox regression, including AC090559.1, AL133371.2, AC096667.1, MROCKI, LNCAROD, and LINC01094. Among the six survival-related lncRNAs, AC090559.1 demonstrated its prognostic value in various programmed cell death [41–44]. AL133371.2 can also predict the prognostic of autophagy-related and necroptosis-related survival in different cancers [45, 46]. LINC01094 was known for their regulatory function in glioblastoma and ovarian cancer [47, 48]. LINC01094 was reported to regulate the ovarian cancer progression via modulation of miR-577 [47]. LINC01094 facilitated cell invasion via targeting miR-532-3p and Wnt/b-catenin pathway in ovarian cancer [49]. Whereas the function of LNCAROD has been studied in other cancers [50, 51], the function of MROCKI and AC096667.1 remains largely unknown, especially in pyroptosis and ovarian cancer.

On top of the two survival-related lncRNAs we selected with multivariate Cox regression, we built a signature model with the riskscore to evaluate the value of the two lncRNAs in ovarian cancer prognostic aspect. The signature model consists of two lncRNAs, MROCKI and LNCAROD, where studies have showed that MROCKI regulates inflammatory gene expression [52], but the function of LNCAROD remains unclear. One study showed that LNCAROD activated glycolysis via promotion of PKM2 expression and facilitated hepatocellular carcinoma malignancy [50]. Another study revealed that LNCAROD can bind with HSPA1A and YBX1 and promoted cancer progression in head and neck squamous cell carcinoma [51]. In addition, LNCAROD enhanced migration and proliferation via targeting miR-181/PROX1 axis in gastric cancer cells [53]. In our study, LNCAROD is a protective factor, where the higher expression of LNCAROD in ovarian cancer patients have a higher survival rate.

We have also performed GO and KEGG enrichment on DEGs of low- and high-risk patients. We found that DEGs are correlated with immune responses, such as humoral immune response in the Biological Process (BP). Mesothelin, a differentiation antigen, is highly expressed in ovarian cancer, which could induce humoral immune response in ovarian cancer patients [54–56]. Pyroptosis is a gasdermin (GSDM)-mediated programmed cell death, and research has shown that therapies via inducing pyroptosis into tumor cells could evoke the immune system to depress the tumor development [57, 58]. In another words, immune system could be triggered by pyroptosis to produce more immune cells to receive better immunotherapy outcomes. Both GO analysis and mechanism of pyroptosis suggest that there is a correlation between immunization and pyroptosis.

In the boxplot of the tumor microenvironment (TME) cell composition, we are able to see that the CD4 memory resting T cells, Macrophages M2 and Macrophages M0 compost most of the microenvironment in ovarian tumor cells [59, 60]. However, none of the immune cells are significantly different in low- and high-risk groups, and it might be due to the insufficient sample sizes. In a nutshell, we have built a signature risk model with two lncRNAs, MROCKI and LNCAROD, which are both not well studied in pyroptosis for ovarian patients. From this study, we suggest that the model could be a prognostic biomarker for treating ovarian cancer. Both MROCKI and LNCAROD act as protective factors in ovarian cancer patients because higher expression of MROCKI and LNCAROD in tumor tissues are associated with the higher survival rate. There are several limitations of the current study. For example, the future work could employ other datasets to test the efficacy of the current model. In vitro experiments are required to determine the functions of MROCK1 and LNCAROD in ovarian cancer cells. In vivo animal models are necessary to validate the role of these two lncRNAs in ovarian cancer development. The association between MROCK1 and LNCAROD and survival and prognosis should be validated in ovarian cancer patients.

## Conclusion

To sum up, our study has investigated the association between pyroptosis and lncRNAs in ovarian cancer. To build on that, we have constructed a risk signature model to evaluate the value of riskscore in predicting the survival of ovarian patients. By stratifying patients into different risk groups, we found the DEGs between the two groups were involved with tumor immunity. We proposed that the two MROCKI and LNCAROD lncRNAs might be a potential therapeutic biomarker for ovarian cancer treatment.

## Data Availability

The data that support the findings of this study are available from the corresponding author upon reasonable request.

## Abbreviations

LncRNA: Long non-coding RNA
DEG: Differentially expressed genes
CDC: Centers for disease control and prevention
PCD: programmed cell death
GSDM: Gasdermin
TCGA: The cancer genome atlas
GTEx: Genotype-tissue expression
PPI: Protein-protein interaction
AUC: Area under the curve
OC: Ovarian cancer
GO: Gene oncology
KEGG: Kyoto Encyclopedia of genes and genomes
GSEA: Gene set enrichment analysis
GSVA: Gene set variation analysis
